# Prioritizing Surgical Care on National Health Agendas: A Qualitative Case Study of Papua New Guinea, Uganda, and Sierra Leone

**DOI:** 10.1371/journal.pmed.1002023

**Published:** 2016-05-17

**Authors:** Anna J. Dare, Katherine C. Lee, Josh Bleicher, Alex E. Elobu, Thaim B. Kamara, Osborne Liko, Samuel Luboga, Akule Danlop, Gabriel Kune, Lars Hagander, Andrew J. M. Leather, Gavin Yamey

**Affiliations:** 1 King’s Centre for Global Health, King’s College London and King’s Health Partners, London, United Kingdom; 2 Global Health Group, Global Health Sciences, University of California, San Francisco, San Francisco, California, United States of America; 3 Department of Surgery, Mulago Hospital, Kampala, Uganda; 4 Department of Surgery, College of Medicine and Allied Health Sciences, University of Sierra Leone, Freetown, Sierra Leone; 5 Department of Surgery, Port Moresby General Hospital, Port Moresby, Papua New Guinea; 6 Department of Anatomy, Makerere University College of Health Sciences, Kampala, Uganda; 7 Department of Clinical Sciences–Lund, Lund University, Lund, Sweden; 8 Duke Global Health Institute, Duke University, Durham, North Carolina, United States of America; Makerere University Medical School, UGANDA

## Abstract

**Background:**

Little is known about the social and political factors that influence priority setting for different health services in low- and middle-income countries (LMICs), yet these factors are integral to understanding how national health agendas are established. We investigated factors that facilitate or prevent surgical care from being prioritized in LMICs.

**Methods and Findings:**

We undertook country case studies in Papua New Guinea, Uganda, and Sierra Leone, using a qualitative process-tracing method. We conducted 74 semi-structured interviews with stakeholders involved in health agenda setting and surgical care in these countries. Interviews were triangulated with published academic literature, country reports, national health plans, and policies. Data were analyzed using a conceptual framework based on four components (actor power, ideas, political contexts, issue characteristics) to assess national factors influencing priority for surgery.

Political priority for surgical care in the three countries varies. Priority was highest in Papua New Guinea, where surgical care is firmly embedded within national health plans and receives significant domestic and international resources, and much lower in Uganda and Sierra Leone. Factors influencing whether surgical care was prioritized were the degree of sustained and effective domestic advocacy by the local surgical community, the national political and economic environment in which health policy setting occurs, and the influence of international actors, particularly donors, on national agenda setting. The results from Papua New Guinea show that a strong surgical community can generate priority from the ground up, even where other factors are unfavorable.

**Conclusions:**

National health agenda setting is a complex social and political process. To embed surgical care within national health policy, sustained advocacy efforts, effective framing of the problem and solutions, and country-specific data are required. Political, technical, and financial support from regional and international partners is also important.

## Abstract 2

Gavin Yamey and colleagues discuss the factors that affect priority setting for surgical care in LMICs and how this important treatment can be prioritised.

## Editors' Summary

**Background:**

Improving human health is a key global concern. Three of the eight Millennium Development Goals agreed to by world leaders in 2000 and designed to eradicate extreme poverty globally by 2015 were directly concerned with public health improvement. And health is central to the Sustainable Development Goals adopted in 2015. But despite health being a global concern, individual countries are largely responsible for addressing the health needs of their populations. All countries have to weigh the health challenges that face their populations and decide which programs and services to prioritize within their national health systems. The allocation of scarce public resources to competing health and other priorities is a complex social and political process, especially in low- and middle-income countries (LMICs). Little is known about why governments channel resources towards some health challenges and not others or about why some health issues become embedded within national health policy while others—including those responsible for a large burden of illness—are largely ignored by national health systems.

**Why Was This Study Done?:**

Surgical care provision is given low priority in the health systems of most LMICs. Only 6.3% of the world’s surgical procedures are undertaken in the poorest countries, where more than a third of the world’s population lives, and most premature deaths from untreated surgical conditions (diseases, illnesses, or injuries in which surgery can potentially improve the outcome) occur in LMICs. Moreover, surgical conditions kill more people every year than HIV/AIDS, tuberculosis, and malaria combined. Understanding why surgical care is a low priority within national health systems in LMICs could provide insights into the social and political processes that drive health agenda setting and resource allocation. In this qualitative case study, the researchers examine the factors influencing the position of surgical care in the national health agendas of Papua New Guinea, Uganda, and Sierra Leone. Although the provision of surgical care has recently improved in Papua New Guinea, all three of these LMICs have a high burden of surgical conditions and inadequate surgical services. A qualitative study examines peoples’ opinions, explanations, and motivations for a particular issue, in order to understand the “why” and “how” of decision-making.

**What Did the Researchers Do and Find?:**

For their study, the researchers used “process tracing,” a qualitative approach that uses two or more methods to analyze change and causation. Specifically, the researchers conducted semi-structured interviews with surgeons, politicians, and other stakeholders to elicit information about how and why different health issues, including surgical care, are prioritized in each study country. They “triangulated” (combined) the information collected in the interviews with information about national health plans and policies and data from country reports and the academic literature. Finally, they analyzed the data using a conceptual framework with four components (actor power, ideas, political context, and issue characteristics) to identify the factors that influence surgical care prioritization. The researchers report that the priority of surgical care varied between countries but was highest in Papua New Guinea. In Papua New Guinea, surgical care was firmly embedded within the health system and received significant domestic and international resources. Notably, three dominant factors influenced whether surgery was prioritized—the level of advocacy by the local surgical community, the national political and economic environment, and the influence of donors and other international actors on national agenda setting.

**What Do These Findings Mean?:**

These findings provide insights into the process of national health agenda setting in Papua New Guinea, Uganda, and Sierra Leone and highlight the complex interplay of social and political factors underpinning this process. In particular, they identify three dominant factors that have influenced whether surgery is prioritized as a health issue in these three LMICs. Notably, the results from Papua New Guinea show that a strong surgical community can generate priority from the ground up, even when other factors are unfavorable for the prioritization of surgical care. These findings may not apply to other LMICS, and certain aspects of the study design may affect their accuracy. For example, the interviewers were surgeons or surgical trainees, which raises the possibility of interviewer bias. Overall, however, these findings suggest that sustained advocacy effort, effective framing of the problem of inadequate surgical care and of the solution to this problem, accurate country-specific data on surgical care indicators, and political, technical, and financial support from regional and international partners will all be needed to ensure that surgical care becomes a priority issue in national health agendas in LMICs.

**Additional Information:**

This list of resources contains links that can be accessed when viewing the PDF on a device or via the online version of the article at http://dx.doi.org/10.1371/journal.pmed.1002023.

- A brief description of the need for global surgery, the provision of high-quality surgical care in all countries, is available
- The World Health Organization briefly explains the need for global surgery and provides links to a research paper that details how people in LMICs are missing out on essential surgery and to a short article on recent initiatives that aim to correct this deficit
- The Lancet Commission on Global Surgery provides information (including videos) on the need for all people to have access to safe, high-quality surgical and anesthesia care, outlines what is needed to achieve global surgical goals, and quantifies the costs of failure if these essential services are not provided
- The Disease Control Priorities project summarizes and synthesizes evidence of the effectiveness of global health interventions and provides comparative economic evaluation of policies to implement those interventions; its 2015 publication Essential Surgery shows that surgical services are among the most cost-effective of all health interventions
- Wikipedia has a page on process tracing (note that Wikipedia is a free online encyclopedia that anyone can edit; available in several languages)
- Brief overviews of qualitative research are provided by Northeastern University and by the University of Surrey

## Introduction

Improving the health of populations, especially those in the world’s poorest regions, is a defining global issue of the early 21st century, attracting significant public and political attention, priority, and resources. Three out of the eight Millennium Development Goals (MDGs), adopted in 2000 by United Nations member states, were directly concerned with public health improvement, and health is central to the Sustainable Development Goals (SDGs) that were adopted in September 2015.

Although health is a global concern, addressing the health needs of populations remains primarily a national responsibility [1]. Faced with a multitude of health challenges, countries respond by allocating resources towards health programs and services within their national health systems [1]. In all countries, but especially in low- and middle-income countries (LMICs), distributing scarce public resources among competing health and development priorities is a complex social and political process [2,3]. There has been relatively little research on why and how different health issues receive different political attention and priority at the national level in LMICs, such that some become embedded within national health policy and some do not [2]. Also poorly understood is why governments choose to channel resources towards some health challenges and not to others, even among diseases that exert a similar burden on the population. These questions are integral to understanding how the health systems, health services, and health policies of countries are shaped.

Surgical care (the provision of operative, peri-operative, and non-operative care, including anesthesia, for all surgical conditions [4]) is almost uniformly afforded very low priority in LMICs [4,5]. Although surgical conditions (any disease, illness, or injury in which surgical care can potentially improve the outcome [4]) claim more lives each year (16.9 million) than HIV/AIDS, tuberculosis (TB), and malaria combined (3.83 million) [6], provision of surgical care is a widely under-recognized health challenge at both national and international levels [4]. Most premature deaths from untreated surgical conditions occur in LMICs [6], where access to surgical care is poor. Only 6.3% of the world’s surgical procedures are performed in the poorest countries, where over one-third of the world’s population lives [7]. Understanding the confluence of factors that may prevent an objectively “high burden” issue such as surgical conditions from being viewed as a priority within national health systems could offer important insights into the social and political processes that underpin agenda setting and resource allocation in health.

Although surgical care attracted little global attention or resources in the period 1990–2010 [8], global interest in surgical care is now rising. Recently, a community of individuals and organizations have come together to advocate for “global surgery” [9], using a variety of high-level channels to promote their cause, including the medical journal *The Lancet* [10], the Disease Control Priorities Project [11], the World Health Organization [12], the World Health Assembly [13], and the World Bank [14]. Though surgery appears to be gaining traction as a legitimate component of global health at an international level, it remains unclear how national health decision-makers view surgical care within their own country health priorities and what effect—if any—early global advocacy efforts have had at the country level. Ultimately, global health goals are realized within nation states. Without a better understanding of the factors that facilitate or obstruct national political priority for surgical care, it will not be possible for the growing global surgical movement to realize the goal of “universal access to safe, affordable surgical care when needed” [4].

We therefore set out to examine facilitating and obstructing factors that influence the position of surgical care on national health agendas in three LMICs: Uganda, Papua New Guinea (PNG), and Sierra Leone.

## Methods

### Ethics

Institutional review board approval was obtained from the Committee on Human Research at the University of California, San Francisco, the PNG National Department of Health Ethics Committee, the Mulago Hospital Research and Ethics Committee, the Uganda National Council for Science and Technology, the King’s College London Research Ethics Committee, and the Sierra Leone Ethics and Scientific Review Committee. We obtained written informed consent from all those who agreed to be interviewed. A unique identifier (e.g., Uganda informant 1, UG01) was assigned for each interviewee to ensure anonymity and protect confidentiality.

### Country Settings

Three case countries were selected from the 139 World Bank–defined LMICs (July 2013 country listings) [15]. In selecting case countries, we sought to include countries at different stages on the pathway to building comprehensive national surgical systems. Potential case countries were evaluated based on objective evidence of key surgical indicators [4], including met and unmet need for surgical care, surgical case volume per population, surgical providers per population, and the presence of a national health plan that specifically included surgical care. Such information was not available for many LMICs, and the case countries selected reflect those for which there were at least some objective country-level data on surgical care.

We then further narrowed down case country selection on the basis of existing professional relationships with investigator institutions and local collaborators, recognizing that access to key actors within each country required drawing on local health, surgical, and political networks. The three case countries selected—PNG, Uganda, and Sierra Leone—represent diverse geographic, political, and cultural settings. Despite the diversity, they share one characteristic: all three countries have poor general health indicators and have experienced documented challenges in providing surgical care to meet population needs (Table 1). These challenges include a high burden of surgical conditions [16,17], low ratios of surgical providers per 100,000 people, low operative volumes [18,19], and inadequate surgical capacity [20–22]. Documented changes over time in these key surgical indicators showed improvement in one case country (PNG), largely unchanged performance in the second case country (Uganda), and worsened performance in the third case country (Sierra Leone).

**Table 1:** Key health and surgical indicators in the case countries and a high-income setting, for comparison.

**Indicator**	**PNG**	**Uganda**	**Sierra Leone**	**United Kingdom**
Population (millions)	7.2	36.3	6.0	64.1
Average life expectancy for males/females (years)	60/65	58/60	45/46	79/83
Infant mortality (per 1,000 live births)	45	38	87	4
Maternal mortality (per 100,000 live births)	215	343	1,360	9
GDP/capita (2014 US dollars)	2,108	696	774	45,603
Total health expenditure per capita (2014 US dollars)	94	59	96	3,598
Total health expenditure (percent GDP)	4.5	9.8	11.8	9.1
Total physicians (per 100,000 persons) (year)	5 (2008)	11.7 (2005)	1.42 (2012)	280 (2014)
Total surgical operations performed yearly^a^ ^,^ ^b^ ^,^ ^c^ (per 100,000 persons) (year)	450 (2015)	~550 (2012)	396 (2012)	8,870 (2014)
Surgical operations performed yearly—public sector^a^ (per 100,000 persons) (year)	450 (2015)	275 (2012)	160 (2012)	8,870 (2014)^d^
Total surgeons^b^ (per 100,000 persons) (year)	1.74 (2015)^e^	0.7–1.0 (2012)^f^	0.97 (2012)^e^	32.4 (2014)^d^

Key health indicators are drawn from the World Bank Group 2014 World Development Indicators [23].

^a^Includes general surgical and obstetric procedures because disaggregated data are not available for all countries.

^b^The Lancet Commission on Global Surgery recommends a minimum global target of least 20 surgical providers per 100,000 population and 5,000 surgical procedures per 100,000 population.

^c^Public sector only; no data available on private sector operative volumes. PNG figures are for general surgical procedures only and do not include obstetric operations, unlike for Sierra Leone, Uganda, and the UK.

^d^These figures are for England only. The total number of surgeons does not include obstetricians as they are represented by a separate college and workforce count in the UK.

^e^Includes surgeons and obstetricians.

^f^Includes surgeons, medical officers, and clinical officers providing surgical care.

GDP, gross domestic product.

Our study explored the differing responses of each country to its unmet surgical need over the past 25 years—spanning the decade prior to the institution of the MDGs and the years after the MDGs were in place. It sought to place these responses in the context of both contemporaneous and historical social, political, and economic events in each country. The differences between case countries allowed us to conduct a comparative analysis of the factors that have facilitated or obstructed surgical care from receiving national political attention, priority, and action.

### Framework for Examining Determinants of Political Priority

To analyze factors influencing priority for surgical care in each of the three case countries, we drew on a previously published conceptual framework developed by Shiffman and Smith (Table 2) [24]. This framework has been used to examine political priority at the global level for maternal mortality, health systems strengthening, mental health, and neonatal survival as well as national political priority for safe motherhood in LMICs [2,24–27]. A health problem is defined as having political priority when (1) political leaders publicly and privately express sustained concern for the problem; (2) political leaders and governments, through an authoritative decision-making process, enact policies that offer widely embraced strategies to address the problem; and (3) financial resources commensurate with the problem’s gravity are mobilized and allocated to address the problem [2,24].

**Table 2:** Conceptual framework for understanding factors shaping political priority for a health issue.

Component	Description	Factors Shaping National Political Priority
**Actor power**	The strength of the individuals and organizations concerned with the issue	1. Policy community cohesion: the degree of coalescence among the network of individuals and organizations that are centrally involved with the issue at the national level 2. Internal actors: the presence of individual leaders and strong champions capable of uniting the national policy community; the effectiveness of national organizations or coordinating mechanisms with a mandate to lead the initiative 3. External actors: the role of international development agencies and funders in promoting and supporting the initiative 4. Civil society mobilization: the extent to which grassroots organizations and electoral players have mobilized to press international, national, and local political authorities to address the issue at the national level
**Ideas**	The ways in which those involved with the issue understand and portray it	5. Internal frame: the degree to which the policy community agrees on the definition of, causes of, and solutions to the problem 6. External frame: public portrayals of the issue in ways that resonate with external audiences, especially the political leaders who control resources
**Political contexts**	The environments in which actors operate	7. Policy windows: political moments when national conditions align favorably for an issue, presenting opportunities for advocates to influence decision-makers 8. Economic environment and support: the existence of national funding systems and mechanisms for health initiatives; the intersection of private business and health 9. National governance structure: the degree to which norms and institutions operating in a sector provide a platform for effective collective action
**Issue characteristics**	Features of the problem	10. Credible indicators: key indicators that show the severity of the problem and its size relative to other problems, and that can be used to monitor progress (e.g., disability-adjusted life years) 11. Severity: the size of the burden relative to other problems as indicated by objective measures 12. Effective interventions: the extent to which proposed means of addressing the problem are clearly explained, cost-effective, backed by scientific evidence, simple to implement, and affordable

This framework has been modified from the original framework of Shiffman and Smith [24] to include factors shaping national political priority that are more specific to surgical care.

The framework identifies determinants of political priority for health initiatives and organizes these into four overarching categories through which to understand the generation of political support and collective action for an issue. These are (1) actor power, (2) ideas, (3) political contexts, and (4) issue characteristics. The cumulative impact of the presence of different factors across these four categories is broadly understood to improve the likelihood that an issue will receive priority [24].

### Process-Tracing Methodology

To examine how surgical care is prioritized at a national level, we used process tracing, a qualitative case study research methodology that is commonly used in political science [28]. Process tracing triangulates multiple sources of information to minimize bias and to identify and test causal mechanisms of a theory. It analyzes change and causation, and focuses on describing and analyzing sequences of independent, dependent, and intervening events or variables [29]. This methodology is unique in its ability to reveal social and political processes within a real-life context, while also accounting for historical influences.

### Data Collection

In each of the three case countries, we conducted interviews with key health and political actors involved in events and processes related to health agenda setting. Using the conceptual framework outlined in Table 2, we developed a semi-structured interview guide with open-ended questions (the guide is in S1 Text). Questions were designed to query the attitudes, values, beliefs, and knowledge of the key informants (KIs) about how and why different health issues, including surgical care, were prioritized within their country.

In total, 29 interviews were conducted in PNG, 32 in Uganda, and 12 in Sierra Leone between March 1 and July 31, 2014, lasting on average 1 h in length. All interviews were conducted in English by A. J. D. (Sierra Leone), K. C. L. (Uganda), and J. B. (PNG), assisted by the local investigators A. E. E., T. B. K., O. L., S. L., A. D., and G. K. The interviews were audio recorded and transcribed. KIs included health care providers (surgical and non-surgical providers from the public, private for-profit, and non-governmental organization [NGO] sectors), health educators and policy-makers, politicians, civil society members, and health funders (S1 Table lists the professions of the KIs). KIs were identified from professional networks, by review of the published and grey literature on surgical care and health agenda setting in each country, and by asking other KIs. We also carried out archival research on the history of health and surgical care within each country, using both peer-reviewed and grey literature. Sources of grey literature included documents published by the government of each country (including the ministry of health [MoH] and the ministry of finance) or NGOs, written accounts of the history and current state of surgery by independent authors, conference presentations, and accounts published by other news outlets.

### Data Analysis

All interviews were transcribed by one of the authors or a commercial transcription service. Transcripts were analyzed with the assistance of Dedoose, a qualitative data analysis and research software program [30]. Each interview was coded independently by one of three authors (A. J. D., K. C. L., or J. B.). A random sample of interviews from each country underwent double-coding, to ensure reliability. Any differences were resolved through discussion between the two coders and, where differences could not be resolved, through adjudication by a third coder. The data analysis process was influenced by a grounded-theory approach [31] to analyze the KI interviews and describe the determinants of national political priority for surgical care in each country. This approach involved reading through the transcript, discussing general themes and concepts, and grouping the concepts into the previously described framework.

During the data analysis process, information on timelines and events revealed through the interviews was triangulated with primary sources, academic and grey literature, and historical accounts. In addition, the local investigators in each country, as well as one independent actor from each country who had a long history of involvement in the health or surgical sector, specifically reviewed the manuscript for historical accuracy prior to submission. Two of these independent actors were also KIs in their respective countries.

### Data Reporting

This study was designed, analyzed, and reported in accordance with the Consolidated Criteria for Reporting Qualitative Studies (COREQ) (interviews and focus groups) [32]. Quotations from individual KIs are used in this manuscript to illustrate and support the broader findings reported in the text.

## Results

### Overall Level of National Priority for Surgical Care

Political priority for surgical care in the three case countries varies, as suggested by our framework criteria and by key objective indicators including surgical provider ratios, operative volume per population, surgical infrastructure, inclusion of surgery within national health policy and plans, and funding flows to surgical care. Priority was highest in PNG, followed by Uganda, and then Sierra Leone.

In PNG, there have been measurable successes in scaling up surgical services over the past 25 years, most notably through the strategic development of a national surgical workforce. This scale-up has been supported by about US$20 million in external financing (provided through the Australian Agency for International Development [AusAID]) (UG28), in addition to domestic line-item funding from the National Department of Health [17,33]. As a result, the number of Papua New Guinean surgeons increased from 12 in 1990 to 90 in 2015 [17,34], and there is now a PNG-trained surgeon at every provincial hospital [35,36]. Surgical care has been explicitly included in the national health plans of PNG since 1990 [35–37]. Surgical case volume remained very low between 1974 and 1990, but then it significantly increased in the late 1990s and 2000s [17]. While PNG has a considerably higher public sector operative volume than Uganda and Sierra Leone, at approximately 450 general surgical operations per 100,000 population in the public sector in 2015, the country still remains well below the international minimum operative volume target of 5,000 operations per 100,000 population outlined recently by the Lancet Commission on Global Surgery [4]. Surgical infrastructure and equipment needs are primarily supported through domestic health budgets, which have explicitly prioritized improving provincial hospitals and ensuring that surgical equipment and supplies are routinely available [36,38].

I think in the last ten years or so, with the help of outside partners, we started training locally, building [surgical] capacity. This raised the level of surgery done in the country. … More priority was given to surgery. (PNG01)

In Uganda, surgery has received a limited amount of policy attention, and the country has struggled to effectively implement improvements in surgical care. Health care reforms undertaken in the country in 1997 did explicitly include development of district-level basic surgical services [39]; however, supporting policies and funding to scale up human resources and improve surgical infrastructure were not developed alongside (UG01–UG04, UG07) [20,40]. In 2000 the Uganda MoH started producing 5-year national health plans, but none of these so far have explicitly included surgical care, aside from a narrow focus on cesarean section as part of maternal health [41–43]. Provision of surgical services and training is only implied as a strategy to reduce the burden of non-communicable diseases (NCDs) and injuries in the most recent Uganda National Minimum Health Care Package, despite the clear role of surgery in the management of both of these major disease groupings [43,44]. Provision of emergency obstetric care, which includes cesarean section, has been a cornerstone of the national health strategy over the past decade [42,43], but this has not resulted in any appreciable flow-on effect or improvements for general surgical services [20,45,46]. In contrast to PNG, Uganda’s major development partners, namely, WHO; the Global Fund to Fight AIDS, Tuberculosis and Malaria; GAVI, the Vaccine Alliance; and the African Development Bank have directed little to no policy or financial support toward surgical services (UG05, UG14, UG20, UG25, UG28, UG31) [47,48]. Surgical case volume remains low, with the estimated number of major operations per 100,000 population performed in the public sector at 155–166 in 1996–1997 and 154–225 in 2010–2012. Over half of the operations undertaken in Uganda in 2014 were performed in the private sector (either NGO or for-profit). The number of Ugandan surgeons has only marginally increased over the past 20 years, from as few as zero surgeons per 100,000 population in a district hospital in 1996 to one or fewer surgeons per 100,000 population in 2012. In many districts throughout Uganda, there are no surgeons who have completed postgraduate training to provide surgical services. Up until recently, postgraduate training in surgery nationally was limited to ten trainees per year. The number of postgraduate training positions in surgery at Makerere University College of Health Sciences has only recently doubled from 20 trainees per year in 2007 to 40 trainees per year in 2011, with the support of an international academic collaborative [49].

If you speak to either [the] Director General of Health Services or the Minister of Health…and you just open the conversation around surgery, it’s just obvious that it’s just not there. It doesn’t find its place into the narrative of what are our burning priorities. (UG31)

In Sierra Leone, surgical care has been absent from the health agenda for many years. Domestic and international health policy and financing in Sierra Leone has predominantly focused on infectious diseases and maternal and child health [50]. Sierra Leone’s Ministry of Health and Sanitation identifies malaria, HIV/AIDS, TB, reproductive health (including maternal and neonatal mortality), childhood diseases, nutrition-related diseases, mental illness, and water-, food-, and sanitation-borne diseases as the country’s main health challenges [50]. One-third (33%) of total health expenditure is spent on maternal health and family planning, 20% on malaria, and 10% on HIV/AIDS and TB [50]. This focus reflects the high burden of infectious diseases and the country’s poor record on tackling maternal mortality [51], but fails to take into account the growing burden of NCDs and injuries. Non-obstetric surgical conditions—predominantly injuries, congenital disorders, and abdominal conditions—account for up to 25% of all deaths according to a recent national population-based survey [16], suggesting that health policy and budget priorities do not always reflect disease burden. The number of Sierra Leone surgeons has remained very low since the civil war ended in 2001, at around ten total, or 0.9 per 100,000 population, most of whom are concentrated in the capital [36]. Operative volumes in Sierra Leone are the lowest of all three case countries and represent some of the lowest rates recorded in the medical literature [4,7,52]. Severe shortages in surgical infrastructure and supplies have been consistently documented over the past decade, including a lack of electricity, running water, oxygen, and fuel at government hospitals [21,53]. Unmet need for surgical care in the country was recently estimated at greater than 90% [18]. Surgical care in Sierra Leone has, however, received considerable international attention by academics and smaller NGOs [16,18,53–56]. In early 2014, there were also modest successes in raising national political attention and resources for the issue of surgical workforce shortages and the need to develop in-country surgical training, but these successes were soon overshadowed by the Ebola outbreak [57,58].

Surgery is ranked very low among health priorities at the ministry [of health] level. (SL03)

### Actor Power

Actor power is strongest in PNG and has been central in driving forward an agenda for surgery at a national political level (PNG12–PNG14, PNG28). Although there is an array of surgical actors in Uganda and Sierra Leone, they are not yet able to achieve the same degree of sustained and cohesive advocacy as in PNG.

Effective advocacy by domestic surgical actors in PNG has occurred as a result of three key factors: the presence of motivated individual surgical leaders with international links; the formation of a large, cohesive surgical community; and sustained access to political leaders involved in health agenda setting (PNG06–PNG08, PNG21, PNG28, PNG29). Informants credited Professor David Watters, the current President of the Royal Australasian College of Surgeons (RACS), who served as the Academic Head of Surgery at the University of Papua New Guinea from 1992 to 2000, as playing a key role in raising the profile of surgical care in PNG in the 1990s, laying the groundwork for subsequent advocacy efforts (PNG01, PNG03, PNG05, PNG08, PNG12, PNG23). Watters—who had trained in Scotland and worked in Zambia, South Africa, and Hong Kong—was recruited to the University of Papua New Guinea as Professor of Surgery by an outgoing international surgeon [37]. Beginning in 1994, he and several Papua New Guinean surgeons established sub-specialty surgical postgraduate programs in the country, training a large number of local surgeons [35]. This scale-up was supported by both national stakeholders, in the form of the PNG National Department of Health, and regional stakeholders, namely, AusAID and RACS. Although both AusAID and RACS had provided some support to PNG in the form of visiting surgical specialists prior to Watters’s arrival, the formation of a formal partnership between the two to deliver postgraduate surgical training was effectively conceived and brokered by Watters [22,37]. RACS went on to win the tender to provide an AusAID-funded program of support to PNG focused on surgical teaching and training. This funding paid for visiting specialists to teach in PNG and for hospital attachments for PNG trainees in Australia. The program also fostered a long-term partnership between the two countries, and their respective surgical communities, focused on surgical capacity building [37]. This partnership has now been active without interruption since 1994 and has resulted in about US$20 million of financial support across this same time period (PNG28) [33].

This concerted effort to scale up surgical training not only substantially increased access to surgical care throughout the country, making it more visible to the public and to policy-makers, but generated a critical mass of surgeons, who coalesced to form a strong collegial network over time (PNG28) [34]. This network was able to communicate effectively with political health leaders via the Chief Surgeon, a National Department of Health position held by a surgeon elected from the PNG surgical community. The Chief Surgeon has facilitated conversation between surgical providers (including representatives from each region) and PNG policy-makers and politicians in the National Department of Health (PNG04, PNG05, PNG19, PNG22).

We have a strong community of surgeons. We have become reliant on each other. The network is very strong. (PNG12)

[Priority for surgical care] is bottom up. It is being driven from ground level. (PNG13)

In contrast, domestic actors in Uganda and Sierra Leone have not been able to raise priority and mobilize resources for surgical care at the national level in a sustained manner. In Uganda, many widely respected surgeons have held, and currently hold, leadership positions that carry political influence (UG03–UG07, UG09–UG11, UG14, UG17, UG18, UG22, UG28). However, they have been variably effective in using their influence to drive forward an agenda for surgical care. From 1992 to 1999, the Uganda MoH had a Chief Surgeon position that provided a formal mechanism for surgeons to influence health policy decisions. During this time, Chief Surgeon Dr. Francis Omaswa led major health sector reforms, including establishing district health centers to provide basic surgical services, such as cesarean sections (UG02, UG04, UG18), which raised priority for surgery within the health system. However, this Chief Surgeon position was dissolved during restructuring in 1999, taking with it a direct conduit into the MoH for surgeons. Since then, surgeons who have found themselves in other government or leadership roles have been unable or unwilling to leverage their positions of influence to promote surgery at a higher political level. In addition, the wider surgical community in Uganda has been heavily clinically focused and has not been as politically active—either individually or collectively—as the network in PNG. For example, the Association of Surgeons of Uganda has struggled to fulfill its mandate to represent the surgical community’s political interests due to high levels of political apathy among its member base (UG07, UG15, UG24, UG31). As a result, surgery has largely disappeared from the national political agenda over the past 15 years.

[Surgeon] influence is at the moment limited. … Truth is, not many doctors are even bothered about [the Association of Surgeons of Uganda’s] existence. … We don’t associate; we are not able to build a critical mass that can lobby. (UG07)

In Sierra Leone, although there are several dedicated individuals working to improve surgical care, no clear domestic leader has emerged to champion the issue at a national political level (SL03, SL05–SL07, SL11, SL12). Many of the country’s surgeons left during the civil war of 1991–2002, and those that remained are nearing or past retirement age. In the absence of postgraduate surgical training in the country, there is no steady influx of new surgical providers into the community to bolster numbers. As a result, the surgical community remains small, lacking the critical mass required for effective collective advocacy. At a regional level, the West African College of Surgeons holds political influence and has played an important role in supporting the domestic surgical community in advocating for postgraduate surgical training in Sierra Leone (SL03, SL09).

A wide array of other international actors is also involved in provision of surgical care and surgical training within the three countries, with varying involvement in advocacy efforts. International actors include WHO’s Global Initiative for Emergency and Essential Surgical Care, international academic partnerships, and surgical NGOs. As most are not large-scale global health funders, they do not have the same level of political influence as the major international actors involved in child health, maternal health, and infectious diseases in each country (e.g., GAVI, the Vaccine Alliance; the US President’s Emergency Plan for AIDS Relief; the Global Fund to Fight AIDS, Tuberculosis and Malaria). Some international surgical NGOs have operated in isolation from local actors, both in terms of the provision of clinical services and in their interactions with local government and policy-makers. This isolation has contributed to fragmentation and at times inadvertently undermined the efforts of domestic actors (UG28, SL05, SL11). However, the presence of other international actors has helped to raise the profile of surgical care (SL11, SL12). For example, Global Partners in Anesthesia and Surgery, an academic alliance active in Uganda over the past decade, has undertaken research highlighting the large unmet surgical and anesthesia need in the country and has used resulting data to support national advocacy efforts (UG10, UG28) [59].

Grassroots civil society engagement with surgical issues is completely absent in all three case countries, and widely viewed as difficult to generate given a history of distrust by citizens in the health system, low levels of education, and poor health literacy (UG07, UG12, UG26–UG28, SL01, SL05, SL06). There is little civil society engagement with other key health issues in these countries, with the exception of HIV/AIDS and, more recently, child and maternal health, TB, and malaria. Low population coverage with surgical services in Sierra Leone and Uganda means that many members of the public will never have contact with a surgical provider [18,20]. In urban areas, where there is greater awareness of surgical care, this service is perceived as a luxury for the wealthy rather than as a requirement of a functioning health system or a basic human right (UG06, UG07, UG12, UG17, UG23, UG26, UG27, SL06, SL11). As a result, there is no public pressure on politicians to address surgical or anesthetic need within their health manifestos.

There’s…no major patient group to my knowledge that is really standing up to say, “We need better surgical care.”… There’s no organized group that’s really pushing down the doors of Parliament. (UG28)

### Ideas: Framing the Problem

Finding a set of ideas that clearly describe the problem of inadequate surgical care and that resonate with the public, policy-makers, and politicians is a major challenge for the three case country surgical communities. While each professional surgical community unites around a shared belief that there is a large unmet need for surgical care, it struggles to articulate this need using a common narrative (PNG01, PNG11, PNG16, UG23, UG25, SL09, SL11). The surgical community in PNG has had measurable success in advocating for surgical care by using a narrow focus on trauma, a high-burden, high-visibility issue for the country that resonates with policy-makers and politicians (PNG03) [36]. Road traffic injuries, violence (including domestic violence and tribal warfare), and self-harm are the leading causes of death for both men and women aged 15–49 years in PNG, and trauma is responsible for 45% of all inpatient surgical costs [60,61]. However, the community struggles to broaden that frame to include other aspects of surgical care, including anesthesia (critical for performing any surgical intervention), cancer, and obstetric surgery (PNG08, PNG10, PNG15, PNG21), which are also high-need areas [60].

In Uganda, surgical care advocates have made multiple attempts at framing the issues surrounding surgery for policy-makers [40]. They have quantified the capacity of Uganda’s public hospital to deliver surgery, connected the issue to achieving the MDGs, framed surgery as an essential part of primary health care or a component of health systems strengthening, and emphasized the high burden of surgical disease in their country (UG01–UG08, UG10, UG28). However, despite these frames, policy-makers remain largely focused on other health issues. The surgical community in Sierra Leone was historically very ineffective in its ability to frame the need for surgical care to domestic political audiences. However, in 2014 it experienced some success, developing a narrative around the need for postgraduate surgical training to overcome a surgical workforce crisis—a narrative that resonated with political leaders (SL06, SL09, SL11).

One of the difficulties the respective surgical communities face is using language and arguments to which policy-makers can relate.

We doctors are not good at convincing politicians, but we have got to learn the arts and skills to talk economics, talk business. We cannot keep talking medicine all along when we are dealing with stakeholders who have very little understanding of how the health system operates. That is the link that is missing, and we need to work hard on that. (PNG05)

The communities also experience challenges in generalizing and humanizing the problem of inadequate surgical care. As a result, the problem of inadequate surgical care does not have strong public and political emotional appeal, compared to issues such as maternal and child mortality (PNG11, PNG19, SL06, SL09).

Alongside challenges with defining the problem of inadequate surgical care, the surgical communities struggle to articulate the specific actions required to address this problem. Without a clear pathway for action, it has been hard for these communities to show policy-makers and political leaders that the problems are surmountable and can be effectively addressed through policy change and resource provision (PNG16, PNG19, PNG20, UG27, UG28, SL06, SL09). Although the surgical community in PNG has enjoyed moderate levels of success in raising the political profile of surgery, advocates lack a unified plan for how to further improve surgical care in the country (PNG01, PNG05, PNG08, PNG10, PNG13, PNG14). Without being able to present a clear “ask” to politicians and policy-makers, the PNG surgical community is not able to fully capitalize on the priority the issue is afforded.

### Issue Characteristics

Several features of surgical care in LMICs make raising priority for the issue challenging. Basic descriptive data, such as the number of surgical operations and their indications and outcomes, are not routinely recorded or monitored at a national level (PNG02, PNG04, PNG07, PNG08, PNG11, SL11, SL12). There are also few credible indicators for surgical conditions that clearly demonstrate the severity of the problem relative to other health issues and that can be easily understood by politicians and the public.

You need to know the statistics, so you know exactly what is going on. Then we understand the enormity of the surgical problem. … For example, what are the challenges? I have this in part, but not in total. (PNG20)

If you gather data in surgery—like so many hernias, so many obstructed hernias—the first issue is a lot of people would not understand the data. And it does not have [the same] impact as maternal and child mortality rate has. (SL09)

Although a lack of credible indicators for surgery at the country level is considered a barrier to raising priority, data on the burden of surgical conditions do exist for each country in the academic literature. A nationally representative household survey estimating the burden of surgical conditions in Sierra Leone published in *The Lancet* in 2012 estimated that 25% of the population had a condition requiring surgical care [16]. However, neither policy-makers nor members of the local surgical community were aware of the study findings. Similarly, a number of studies quantifying surgical capacity have been undertaken in Uganda and Sierra Leone [19–21,53], but the results have not been widely used by domestic actors in their advocacy efforts (UG19, SL03, SL06, SL09). There are challenges to distributing knowledge between academic, clinical, and policy circles, especially when the data are generated by international groups (SL02, SL03, UG19, UG28). In contrast, locally generated data appear to be more effectively used by domestic advocacy groups [22]. For example, surgeons in PNG regularly audit the volume of surgical procedures performed in the country’s provincial hospitals and the burden of different conditions (PNG04, PNG08, PNG13–PNG15). These data are regularly provided to the National Department of Health in efforts to engage policy-makers.

Another major challenge the surgical community faces in each country is convincing policy-makers and politicians that surgical care is cost-effective and affordable (PNG07, PNG19, PNG20, UG06, UG07, UG10, UG19, UG25, SL06, SL09, SL11). Country-specific data that would help make this case are not readily available. For example, there are insufficient data on the unit costs of providing surgical procedures; the infrastructure, equipment, and supplies required to deliver surgical care; the number and type of operations required annually; and the costs of training new surgical providers or building new surgical facilities.

Today, we do not know how many blades are being used, how many catguts and vicryls are being used, what is the usage rate for all of the anesthetic drugs? Somebody needs to tell us. We [need] to know exactly what the unit cost is to deliver a service. (PNG07)

Even where locally generated data outlining the burden imposed by a health issue are available, there is often a disconnect between collecting data and using data to inform health policy.

In the Ministry of Health there is a planning division. They publish data, but I haven’t seen the data being used to plan the policies. So gathering data alone is not the problem, you have to use the data to get action. (SL09)

The significant health systems requirements for delivering effective surgical care are another barrier (PNG03–PNG05, PNG11–PNG14, PNG17, PNG18, PNG23, UG06, UG07, UG28, SL06, SL11). Improving surgical care requires capital investment in infrastructure, equipment, and human resources, which is complex to conceptualize, plan, and implement and cannot be realized over short timescales. All of these factors act as deterrents for policy-makers and politicians, especially those facing short electoral cycles (PNG01, PNG10, PNG12, PNG19). Unlike infectious diseases, there are no “quick fixes” or “magic bullets” for surgical diseases. Providing anti-malarials or anti-retrovirals through vertically delivered programs is simpler and faster to implement and therefore more politically attractive (UG06, UG07, UG28, SL06, SL11). Several informants also stated that a functioning health system is a critical first step before considering scale-up of surgical care, yet health systems are weak in all three countries, and health resources still predominantly flow to disease-specific programs rather than to broader health systems strengthening (UG23, UG25, SL05, SL06, SL09).

I think that’s one of the challenges to developing surgical care. Vertical initiatives are easier to implement from a planning perspective because they can focus on a single disease, and when you talk about surgical care you really are talking about system building and human resources and infrastructure and training. You know it’s more horizontal; you have to develop a whole health system. (UG28)

### Political Contexts

The political environment in which surgical advocates have operated in the three case countries has not been favorable for raising priority for surgery. The overall political focus in health in all three countries is heavily geared towards achieving the health-related MDGs, namely tackling child mortality (MDG 4), maternal mortality (MDG 5), and infectious diseases (MDG 6) (PNG09, PNG11, UG01–UG03, UG05–UG08, UG23, UG25, SL02–SL12). Although access to surgical care is required to reduce maternal mortality (e.g., cesarean section for obstructed labor) and child mortality (e.g., congenital conditions) and to manage complications of infectious diseases (e.g., typhoid bowel perforations), the surgical communities have been unable to effectively link the need for surgery to any of the health-related MDGs at a political level. As a result, surgery has not benefitted from the high level of domestic attention and resources channeled towards the health MDGs, which has largely gone to community-based health strategies rather than hospital-level care.

The MDGs definitely influenced health priorities for politicians. Most of the head policies and most of what money was thrown into was geared to satisfying the MDG maternal and child health goal. Other areas had to be forgotten about. (SL09)

Partly as a result of the sustained focus on the MDGs, very few policy windows (defined as favorable confluences of events providing an opportunity for advocates to press political leaders [24]) have emerged through which the surgical communities could influence decision-makers (PNG12, UG01, UG04, SL03, SL06). Several focusing events have drawn political attention to the issues of inadequate surgical care in Uganda and Sierra Leone but have failed to impact policy formation. These events included international surgical conferences hosted within the countries and attended by local policy-makers and political elite, such as the Bellagio Essential Surgery Group meeting in Kampala, Uganda, in 2008 [62] and the Lancet Commission on Global Surgery meeting in Freetown, Sierra Leone, in 2014 [4]. Although the local surgical communities made concrete attempts to use these opportunities to push forward their agendas in front of a political audience, they were unable to effect policy change. In Uganda, this inability was attributed by some informants to a lack of sustained advocacy efforts as well as a lack of genuine political engagement with the issues (UG7, UG15, UG19, UG28). In Sierra Leone, although political commitments to improve resources for surgical care were given following the Lancet Commission meeting, the Ebola crisis erupted shortly after, and these commitments were put on hold indefinitely.

Efforts to embed surgical care within the national health agenda in each of the case countries are also hampered by a lack of political stability, accountability, and transparency (PNG06, PNG09, PNG12, PNG13, UG03, UG06, UG12, SL02, SL03, SL11). Rapid turnover of high-level MoH staff in Uganda and Sierra Leone means health agendas and policies frequently change, making it hard to build momentum for surgical policy within the ministries (UG04, UG33, SL03, SL11).

We have been dealing with four Ministers of Health since we’ve been here, and we see that when the new one comes in, they haven’t heard of surgical care at all. … So we feel that we are sort of erasing, starting the same kind of thing and building up our case…again. (SL03)

Informants also believed policy setting and resource distribution for health are heavily influenced by personal and partisan politics, cronyism, and corruption, rather than by objective evidence or community lobbying (PNG01–PNG05, PNG12, UG05, UG06, UG23, UG25, SL02, SL05, SL06, SL10). Even when compelling country-specific data on surgical need are available, building a rational, evidence-based case for surgical care is usually not sufficient to influence decision-makers to act, particularly in countries with low levels of political accountability (PNG12, UG06, SL02, SL05).

I mean politicians will go and will dictate where money is spent. They will allocate to whatever they want to use as priority areas. And they will give to their projects—whatever they think is helpful for their political ambitions and whatever is likely to secure their political agenda. (PNG12)

There’s gross abuse, I mean this is no secret, gross abuse of the budgetary process and expenditures in health. These monies find ways into peoples’ pockets or activities that benefit individuals that do not benefit the health care system. (UG06)

In PNG and Uganda, personal political interests have typically worked against surgery (PNG01–PNG05, PNG25, UG02, UG05, UG06, UG17, UG28). However, personal interest from high-level leaders also has the potential to elevate surgical issues to high priority status, despite years of unsuccessful attempts to generate support through official channels (e.g., within the MoH) (PNG19, PNG25, UG02, UG04–UG07, UG16, UG28). This elevation of priority occurred recently in Sierra Leone when the country’s president took a personal interest in the issue of postgraduate surgical training and was able to quickly mobilize resources to push forward the issue (SL06, SL07, SL09, SL11). Senior surgeons in Sierra Leone, together with surgeons from international partnering institutions, made a personal appeal to the president on three occasions in early 2014 to replenish a depleted and aging surgical workforce (in June 2014, the country had only ten surgeons, and most were nearing retirement). They also used neighboring Liberia as an example to show that establishing postgraduate surgical training was possible, thereby also engendering a sense of regional competition, something observed as being a strong political motivator for the issue of maternal mortality in Sierra Leone.

Even if you go to the minister with the best story in the world, the minister is cash strapped. Or they say, “Ah, we can’t do this, there are other priorities.”… But if the president says this is a priority and this has to be done, he is able to knock heads together. (SL06)

The domestic political environment for health is also heavily influenced by the agendas of international donor organizations (PNG01–PNG16, PNG19, PNG20, PNG22, PNG26, PNG27). Over the past decade, most major international donors have prioritized the health-related MDGs and have channeled resources accordingly (UG02, UG03, UG05–UG07, UG20, UG31, SL01–SL03, SL06, SL07, SL10). These organizations exert significant political influence as they make substantial contributions to the overall health budgets—and the economies—of the three countries. For example, in Sierra Leone, international aid represents 20% of the country’s income [63]. In Uganda, off-budget donor health funding (funding that falls outside of the government budget and is directly channeled to ministries or programs) amounts to 40% of the total health budget and is almost exclusively channeled to HIV/AIDS, malaria, and TB [64]. Surgery is not a priority for the major donors [4,65], and therefore there is no external pressure on policy-makers and politicians to prioritize surgical care in the same way that there is for HIV/AIDS, malaria, TB, and maternal and child health.

I think the reason why these things were not prioritized was because we have so come to depend on international donors, so what the donors didn’t fund, we didn’t look at. (SL06)

International donors also channel much of their funding towards vertically orientated projects and targets (UG06, UG07, UG23, UG25, UG28, SL03, SL05, SL06, SL11). Informants believed that this negatively impacts the development of the overall public health system, including the maintenance and development of hospital-based services for surgery, especially in Sierra Leone and Uganda. Donor funding also heavily influences the distribution of human resources for health in a way that can hinder national surgical care. Doctors and health workers have moved away from their public clinical practice to work for organizations and projects that the international community funds, namely, those in the fields of public health, infectious diseases, and maternal and child health (UG02, UG03, UG10, SL06, SL11, SL12). These jobs are better paid than government jobs and are seen as prestigious.

Human resources tend to follow the money. (SL06)

However, in PNG, the development of a strong surgical community and a clear surgical training pathway, which trainees are proud to be associated with, has prevented much of this internal migration away from surgery (PNG06, PNG17, PNG21, PNG28, PNG29). External financing for the scale-up of surgical services in PNG, as well as technical assistance and training from the donor community, played a critical role in the initial development of surgical capacity. However, the development of surgical capacity has also been accompanied by strong signs of domestic political commitment and support for surgery (PNG01, PNG03–PNG05, PNG12, PNG23, PNG29). For example, Papua New Guinean surgical residents are now trained entirely by local surgeons through established national sub-specialty training programs, while surgeons and anesthetists within PNG perform important research related to surgical care without assistance from foreign actors.

In addition to an unfavorable political environment for surgery, the socioeconomic conditions in all three countries have profound effects on agenda setting for health, including priority for surgical care. Low domestic revenues and multiple urgent and legitimate competing development concerns create major distributive challenges for governments in all three countries. Within this highly constrained environment, specific health issues such as surgical care struggle to compete for political attention and resources. Health spending as a proportion of gross domestic product (GDP) varies according to the level of priority it is afforded by governments. For example, in Uganda health ranks behind defense, infrastructure, agriculture, and education in terms of budgetary priorities (UG02, UG06, UG14, UG17) [48,66]. All three case countries consistently spend less on health care than the recommended 15% of GDP set by the Abuja Declaration in 2001 [67]. Out of the three countries, PNG allocates the lowest percentage of its GDP to health (4.5% compared to 9.8% in Uganda and 11.8% in Sierra Leone), despite having the greatest objective success in scaling up surgical care. However, total health expenditure per capita (in 2013 US dollars) is similar between the countries: $94 in PNG, $96 in Sierra Leone, and $59 in Uganda, compared to $3,598 in the United Kingdom and $9,146 in the United States. Much of the financing for PNG’s surgical scale-up has been external, through development assistance given by AusAID. In Sierra Leone, a series of political and health crises over the past 25 years have further derailed progress in health, and have heavily impacted surgical care in the country (SL01–SL04, SL06, SL11) [68]. These include the civil war of 1991–2002 and the recent outbreak of Ebola (which reached its peak after this study was conducted) that killed thousands of people, including 20% of the country’s surgeons [57], crippling the country’s already weak health system and severely impacting surgical volume [58]. These events have at times diverted domestic and international political attention and resources away from longer-term development needs, including surgical services.

## Discussion

### Factors Shaping Political Priority for Surgery in the Three Case Countries

Our analysis identifies three dominant themes that have influenced whether surgery is prioritized as a health issue at the national level in PNG, Uganda, and Sierra Leone. These are (1) the degree of sustained and effective domestic advocacy by the local surgical community, (2) the national political and economic environment in which advocacy efforts take place and health policy setting occurs, and (3) the influence of international actors on agenda setting, especially the relationship between international donors and the ability of countries to determine their own health agendas.

A strong, cohesive, and sizeable group of domestic advocates, predominantly surgeons, has been essential for raising priority for surgical care in PNG from the ground up. Domestic actors were most effective in their advocacy efforts when they were supported by a committed group of regional or international actors, such as regional professional colleges and international development agencies. These regional and international actors served as long-term partners and had the ability to mobilize financial and technical resources to support local efforts. Conversely, poor community cohesion between domestic actors and a lack of sustained international financial and technical support weakened advocacy attempts in Sierra Leone and Uganda.

The national political and economic environment in all three countries has been unfavorable for raising priority for surgical care. Major distributive challenges coupled with a heavy focus on infectious disease, child health, and maternal health have made it difficult for other health issues including surgical care to attract attention and resources. Priority for health issues and health policy setting is often influenced by personal interests or partisan politics, rather than by evidence, making it more challenging to build an effective political case for surgical care.

International actors play a major role in setting global norms regarding priority health issues and targets for LMICs, as evidenced by the heavy MDG focus in all three countries. These actors also significantly influence agenda setting through the large financial contributions they make to domestic health budgets and through the mechanisms by which they channel resources. A lack of emphasis and funding for surgical care at the international level therefore appears to have heavily contributed to the low priority surgical care is afforded within domestic health agendas. Conversely, international funding from AusAID for a locally initiated and driven postgraduate surgical training program has been crucial to the successes seen in PNG, alongside sustained domestic budget commitment from the PNG National Department of Health. While external funding was a crucially important factor in the scale-up of surgical services in PNG, domestic political commitment means that the surgical system would now be robust enough to function largely without outside funding. Surgical care, training, and research are now firmly embedded within national processes.

### Opportunities for Increasing Priority for Surgery

With the recent adoption of the SDGs by United Nations member states, the local and global health landscape is currently undergoing a transition. As a result, new opportunities are emerging through which to raise the priority afforded to surgical care at a national level in LMICs, including in the three case countries. The SDG era appears to be ushering in a new focus on universal health coverage and the strengthening of public health systems and health services, as reflected in the final text of the SDGs [69]. Global efforts to raise priority for surgical care as an integral component of a functioning health system in LMICs have gained momentum. For example, in May 2015 the World Health Assembly passed a resolution supporting the need for access to essential and emergency surgical care as an integral part of universal health coverage [13]. This resolution has the potential to create policy windows at the national level for the surgical community that have been absent during the MDG era. Regional and international actors can provide important political, technical, and financial support for surgical care at the country level and can support local actors in creating and taking advantage of these policy windows. Experiences in the three case countries suggest that the agenda must be locally driven and owned, however, to bring about sustained improvements. Local ownership of both the problems and the solutions is a crucial factor in shaping policy.

The Ebola outbreak in West Africa, which occurred after the interviews for this study were conducted, has also focused global attention on the need to develop resilient health systems in LMICs. The fragility of Sierra Leone’s health system, and especially its hospital-based services, was exposed during the outbreak, and, consequently, strengthening the public health system has been identified nationally and internationally as central to post-Ebola recovery [70,71]. There has been significant mobilization of technical and financial resources by the international community to support such systems strengthening in Sierra Leone [72], presenting a clear opportunity to improve surgical care and related services in the country. National and international interest in regenerating the surgical workforce in Sierra Leone has also increased as a result of the Ebola crisis, following the high-profile loss of the country’s youngest surgeon to Ebola in November 2014 [57].

In addition, LMICs are undergoing an epidemiological transition, with a rising burden of NCDs and injuries [73,74]. Political concern about the health and economic impacts of NCDs and injuries presents a window of opportunity for the surgical communities in each country to demonstrate the need for surgical care in tackling this disease burden. As new national health policies are formed aimed at curbing and treating NCDs and injuries, focus should be placed on embedding surgical care within these policies.

The experience of PNG shows that even in the face of low levels of national political interest, it is possible to influence health agendas and mobilize resources for surgical care from the bottom up. Domestic actors can strengthen their surgical advocacy efforts by developing strong and cohesive national networks and by working with committed regional and international partners to collectively push forward a common agenda. These efforts can be supported through the collection and local dissemination of country-specific indicators that outline the burden of surgical conditions and the health and economic impacts and costs of scaling up surgical care in a way that can be easily understood by the public and politicians. Robust evidence also needs a human face, and effectively framing the issues such that they have strong emotional appeal at a political level can generate substantial traction. Among smaller surgical communities, strategic, well-defined “asks” of policy-makers and political leaders (e.g., establishment of a postgraduate surgical training program in Sierra Leone) are more likely to be politically tractable, financially feasible, and therefore adopted into policy. The Lancet Commission on Global Surgery advocated the development by each country of a national surgical plan [75] that addresses surgical infrastructure, workforce, service delivery, financing, and information management needs, as an important policy document for advancing surgical care within national health agendas [10]. Certainly, explicit inclusion of surgical care within the 5-year national health plans of PNG has ensured that the issue remains on the domestic health agenda and receives ongoing technical and financial resources.

A better understanding of the opportunities to prioritize surgery on the agenda of LMICs can also come from comparing our study results with those of other studies that examined national advocacy for a specific health issue. To give one example, Smith and colleagues recently examined why neonatal mortality had been prioritized or neglected in Bolivia, Nepal, and Malawi, three countries with high neonatal mortality but very different levels of prioritization [76]. Since 2000, neonatal mortality has steadily risen to the top of the national agenda in Nepal; in contrast, attention stagnated then grew in Malawi and grew then stagnated in Bolivia. The factors associated with successful advocacy were “(1) advancing solutions with demonstrated efficacy in low-resource settings, (2) building on existing and emerging national priorities and (3) developing a strong network of domestic and international allies.” Thus, for domestic surgical communities in LMICs, demonstrating efficacy and cost-effectiveness, showing how scale-up of surgical services would build on existing and emerging priorities (especially NCDs and injuries), and forging networks of allies could all provide opportunities for agenda setting. Indeed, a contemporary study by Shiffman showed how the emergence of networks of actors was critical for the rise of newborn survival on the global agenda [27].

### Strengths and Weaknesses of the Study

We believe that our study has five key strengths. First, to the best of our knowledge, this is the first study to examine political priority for surgery at the country level in LMICs, as well as the factors that may explain the prioritization or neglect of surgery as an issue on national health agendas. A recent study by Shawar and colleagues examining the positioning of surgery on the global agenda argued that national-level studies are needed because “national-level dynamics are crucial for generation of political priority for surgery” [77]. Our study provides new information and insights into the complex internal and external processes that influence health agenda setting in LMICs; these insights could help to guide future policy making at both the national and global level. Second, the inclusion of three countries that are at differing stages on the pathway to building a comprehensive national surgical delivery system allowed us to compare and contrast factors that have facilitated or obstructed priority for surgery between countries. Third, our study included a highly diverse range of KIs in each country, including surgeons; anesthetists; allied health workers; surgical, anesthesia, and obstetric trainees; medical educators; hospital administrators; MoH officials; representatives of NGOs and bilateral and multilateral organizations; and politicians. This meant that we could gather perspectives from across multiple sectors of the health system. Fourth, by conducting a very large number of interviews, we were able to reach theoretical saturation (defined as “the phase of qualitative data analysis in which the researcher has continued sampling and analyzing data until no new data appear and all concepts in the theory are well-developed”) [78]. Lastly, we took steps to maximize the reliability of our findings by double-coding a random sample of the results.

Despite these strengths, our study has a number of limitations that are common to all qualitative studies. In particular, the findings from the three countries included in our study may not be generalizable to other national settings. Although common themes emerged from the case countries, priority setting processes are also likely to be dependent, to at least some extent, on national contextual factors. Another limitation facing qualitative researchers is that they can bring their own “interviewer bias” to the study. In our case, the interviewers were all surgeons or surgical trainees who believe that surgery should have a higher priority in LMICs, a view that could have influenced the interview process. We took steps to reduce this bias by using a semi-structured interview guide that had been carefully designed to avoid leading questions. The three case countries were selected on the basis that data on key surgical indicators in the country were available and that existing professional relationships and collaborations existed that facilitated access to a broad range of KIs in each country. These selection criteria could represent a potential source of bias and may further limit the generalizability of the study findings. Finally, although we reached theoretical saturation on analysis of data from all three countries, the number of KIs in Sierra Leone was substantially smaller than in Uganda and PNG. This smaller number in part reflects the smaller size of the overall population as well as the health and surgical communities in Sierra Leone. However, the final number of KIs in Sierra Leone was also impacted by the fact that the period during which the interviews were conducted in Sierra Leone (June–July 2014) overlapped with the onset of the Ebola outbreak. Specifically, two identified KIs were unable to provide an interview as previously planned owing to their heightened responsibilities and the subsequent time constraints imposed by coordinating the initial outbreak response. Interviewing these individuals after the outbreak had subsided was not deemed appropriate given the large time period that had elapsed and the significant changes that had occurred in the health sector as a result.

### Conclusions

National health agenda setting is a complex social and political process, especially in LMICs facing major distributive challenges coupled with low levels of political accountability. Domestic health agendas and policies remain heavily influenced by international donor priorities and funding and may be only weakly informed by evidence of local disease burden or health need. Challenges with public and political framing of the need for surgical care have prevented it from receiving greater priority, as has the absence of impactful, country-specific surgical indicators. Development of cohesive domestic surgical communities with a critical mass of members appears to be important for sustained, effective surgical advocacy. Domestic advocacy efforts can be enhanced by political, technical, and financial support from committed regional and international partners. For example, Rwanda’s domestic support for surgery is being enhanced by the donor-funded Human Resources for Health Program, which includes funding for surgical training [79]. Embedding surgical care explicitly within long-term national health plans is important for ensuring sustained political attention and resource allocation. Although the political context in LMICs has not been favorable for surgical care over the past two decades, the post-2015 health landscape is poised to present several opportunities for surgical care to ascend as a priority issue on national health agendas.

## Supporting Information

S1 Table List of the professions of the key informants. (DOCX)

S1 Text Semi-structured interview guide. (DOCX)

## Abbreviations

AusAID: Australian Agency for International Development
GDP: gross domestic product
KI: key informant
LMICs: low- and middle-income countries
MDG: Millennium Development Goal
MoH: ministry of health
NCD: non-communicable disease
NGO: non-governmental organization
PNG: Papua New Guinea
RACS: Royal Australasian College of Surgeons
SDG: Sustainable Development Goal
TB: tuberculosis
