# Supplementary material for: Prioritizing Surgical Care on National Health Agendas: A Qualitative Case Study of Papua New Guinea, Uganda, and Sierra Leone
Source: PLoS Med. 2016 May 17;13(5):e1002023. doi: 10.1371/journal.pmed.1002023 (PMC4871553; doi:10.1371/journal.pmed.1002023)
Supplement: S1 Table — (DOCX) [file pmed.1002023.s001.docx]

S1 Table. List of the professions of the key informants

Papua New Guinea

| PNG1 | Hospital Administrator |
| --- | --- |
| PNG2 | Policy Maker |
| PNG3 | Surgeon |
| PNG4 | Surgeon |
| PNG5 | Hospital Administrator, Surgeon |
| PNG6 | Physician in Private Practice |
| PNG7 | Hospital Administrator, Obstetrician and Gynecologist |
| PNG8 | Anesthetist |
| PNG9 | Medical Educator, Hospital Board Member |
| PNG10 | Medical Educator |
| PNG11 | Medical Educator, Surgeon |
| PNG12 | Hospital Administrator, Surgeon |
| PNG13 | Surgeon |
| PNG14 | Surgeon |
| PNG15 | Hospital Administrator |
| PNG16 | Policy Maker |
| PNG17 | Policy Maker, Anesthetist |
| PNG18 | Physician |
| PNG19 | Policy Maker |
| PNG20 | Policy Maker |
| PNG21 | Medical Educator, Obstetrician and Gynecologist in Private Practice |
| PNG22 | Policy Maker |
| PNG23 | Medical Educator, Surgeon |
| PNG24 | Obstetrician and Gynecologist |
| PNG25 | Policy Maker |
| PNG26 | Medical Educator, Physician |
| PNG27 | Medical Educator, Physician |
| PNG28 | Medical Educator, Physician |
| PNG29 | Medical Educator, Surgeon |

Uganda

| UG01 | Former Professional Surgical Association Leader |
| --- | --- |
| UG02 | Regional Hospital Administrator |
| UG03 | Surgeon in a Regional Referral Hospital |
| UG04 | Tertiary Hospital Administrator |
| UG05 | Non-Ugandan Surgeon, Educational Partner |
| UG06 | Former Ministry of Health Official, Public Health Academic |
| UG07 | Professional Surgical Association Leader |
| UG08 | Tertiary Hospital Administrator |
| UG09 | Former Regional Surgical College Leader |
| UG10 | Academic Anesthesiologist |
| UG11 | Ministry of Health Official |
| UG12 | Civil Society Member |
| UG13 | Ministry of Public Service Official |
| UG14 | Ministry of Health Official |
| UG15 | Professional Medical Association Leader |
| UG16 | Oncologist |
| UG17 | Ministry of Health Official |
| UG18 | Former Ministry of Health Official |
| UG19 | Academic Surgeon |
| UG20 | Development Partner |
| UG21 | Medical Educator, Obstetrician and Gynecologist |
| UG22 | Ministry of Health Official |
| UG23 | WHO Local Official |
| UG24 | Surgeon Trainee |
| UG25 | WHO Local Official |
| UG26 | Civil Society Member |
| UG27 | Civil Society Member |
| UG28 | Non-Ugandan Surgeon, Educational Partner |
| UG29 | Tertiary Hospital Administrator |
| UG30 | Tertiary Hospital Administrator |
| UG31 | Development Partner |
| UG32 | Previous Hospital Administrator |
| UG33 | Non-Ugandan Provider, Educational Partner |

Sierra Leone

| SL01 | Hospital Administrator, NGO |
| --- | --- |
| SL02 | Medical student |
| SL03 | Clinician, NGO |
| SL04 | Surgeon, Medical Educator |
| SL05 | Surgeon |
| SL06 | Anaesthetist, Professional Medical Association Leader |
| SL07 | Surgeon |
| SL08 | Ministry of Health Official, Policy maker |
| SL09 | Physician, Private Sector |
| SL10 | Obstetrician & Gynaecologist |
| SL11 | Hospital Administrator, Tertiary Government Hospital |
| SL12 | Medical Officer in Surgery, District Hospital |
