# Supplementary material for: Prioritizing Surgical Care on National Health Agendas: A Qualitative Case Study of Papua New Guinea, Uganda, and Sierra Leone
Source: PLoS Med. 2016 May 17;13(5):e1002023. doi: 10.1371/journal.pmed.1002023 (PMC4871553; doi:10.1371/journal.pmed.1002023)
Supplement: S1 Text — (DOCX) [file pmed.1002023.s002.docx]

**S1 Text. Semi-structured interview guide**

**General**

- What are the main health care priorities in [country] currently at a national level?
- Why have these areas received attention do you think?
- What are the key priorities in [country] in health at present in your own opinion?
- How does surgery fit or not fit in with the key health priorities for [country]?
  - Why is this?
- Do you think that the ministry of health and the ministry of finance correctly aligns their policies and funding with the priority health areas in [country]?
- If you could double your health budget (or for local informants, your hospital budget) from next year, where would you spend the additional money?
- How does surgery fit or not fit in with the key health priorities in [country]?
  - Why is this?
- What is the current priority for scale-up of surgical capacity in the health agenda of [country]?
  - How important is providing surgery compared to other health initiatives such as preventing HIV or decreasing maternal deaths?
- Please tell us about your country’s (or province’s) major successes and challenges with providing surgery.
- How do you see surgery contributing to these priorities and helping to achieve your country's health goals?
- What is required to see surgery as a greater priority to your healthcare system?

**Actor Power**

- Who is responsible for setting major national health policy and who holds significant influence over these decisions? Both within the country and internationally?
  - Is there agreement over the general direction in which to invest in the health sector?
- To what extent have the health priorities in your country been influenced by;
  - Locally articulated health needs? (i.e. through local advocacy, local and national politics etc)
  - Donor driven agendas (public, private, NGO, bilateral, multilateral etc)
  - The MDG agenda
  - Others?
- What sources within the country, if any, have provided pressure on policy makers to increase surgical capacity? Internationally?
  - How effective have they been in achieving their goals?
  - How do you regard and utilize the WHO recommendations for Emergenyc and Essential Surgical Care (EESC)? How do these recommendations support or differ from how you would like to see surgery implemented within your own country?
  - How are local and national leaders similar or different in their roles for promoting surgery?
- Are there any points of cohesion between internal actors and external actors in surgery?

**Ideas**

- Internal Frame
  - What is your definition of essential and emergency surgery?
  - What key surgical interventions are most important for your country?
  - What are the major surgical diseases in this country?
    - *If subject does not see certain issues as ‘surgical’* – Why do you not consider [issue] as a surgical issue / disease? (maternal/perinatal issues for example)
  - How does the community view surgery's role in overall health system strengthening?
  - Can surgery be provided in this country at a low cost?
  - Who should provide surgery or anesthesia? / what is your opinion on the role or need for task shifting in [your country]?
    - Should MDs or surgical technicians be providing surgery and anesthesia??
    - Is there a role for surgical technicians in your country?
- External Frame
  - How should the issue of providing surgery be framed to political leaders in order to generate political support?

**Political Contexts**

- Is providing surgery on the national or local or hospital health agenda in your country?  How did it get there?
- How is health care funded within your country?
  - Public/private/NGO sectors?
  - Out of pocket payment
- What role does each sector play in providing healthcare (or surgery) in your country?
- How much of the health budget, if any, is explicitly allocated toward providing surgery in your country / hospital?
- Have there been any opportunities you are aware of within the national or local political/health agenda when surgery may have gained more traction? e.g. during a focus on maternal health?
  - Was this opportunity utilised? Why/why not?
  - Who drove this or should have driven this in your opinion?
- Are there mechanisms for advocating for health issues/priorities at a local and national level political level?
  - What are these?
  - Are they effective in bringing about change (responsiveness)? Why?/Why not?
  - How could they be more effective?

**Issue Characteristics**

- To what extent are you able to use local and national health data to inform planning and resource allocation in health?
- What tensions exist between pursuing short and long-term strategies in building surgical capacity?
- Identify the major points of intervention for increasing surgical capacity in (your country or local setting)
- How are health priorities addressed in your country? Vertical vs. diagonal vs. horizontal strategies?
- What, if any, are the main health indicators you focus on?
  - Are any of these surgical?
- Are you aware of the burden of surgical disease in your country?
  - Trauma/injuries?
  - NCDs
  - Maternal health
  - Where do these surgical diseases stand in relation to infectious diseases?
- What would you consider the top 10 diseases in your country (rank them)?
- What are the most significant surgical diseases in your country?
  - How important is providing subspecialty surgery in your country?
- What do you think are the main reasons why improving surgical capacity is not given high priority?
- What are the barriers to deliver surgical care in your country?
- How do the requirements needed for delivering surgical services in your country differ from those of infectious diseases, like HIV?
  - What are the infrastructure requirements and gaps?
- Who should be providing surgery?
  - What are the barriers or bottlenecks to adequate human resources for surgery in [your country]?
